# Permanent pacemaker implantation rates following cardiac surgery in the modern era

**DOI:** 10.1007/s11845-020-02254-y

**Published:** 2020-05-22

**Authors:** Jason Kho, Adam Ioannou, Katie E. O’Sullivan, Mark Jones

**Affiliations:** 1 Wexham Park Hospital, Frimley Health NHS Foundation Trust, Wexham, UK; 2 Royal Free Hospital, Royal Free London NHS Foundation Trust, London, UK; 3 Royal Victoria Hospital, Belfast Health and Social Care Trust, 274 Grosvenor Road, Belfast, BT12 6BA UK

**Keywords:** Cardiac surgery, Cardiology, Modern era, Permanent pacemaker

## Abstract

**Aims:**

The aim of this study was to evaluate the incidence of permanent pacemaker (PPM) implantation after cardiac surgery in our institution and investigate risk factors for PPM dependency to provide patients with accurate incidence figures at the time of consent for surgery.

**Methods:**

Data was collected retrospectively from a single tertiary institution from October 2018 to April 2019 inclusive of 403 patients. Incidence of PPM implantation after various cardiac operations was evaluated. A univariate analysis was carried out to identify the independent risk factors related to PPM implantation.

**Results:**

Ten patients required a PPM (2.48%). The most common indication for PPM implantation post-cardiac surgery was complete heart block (*N* = 7, 70%) followed by bradycardia/pauses (*N* = 2, 20%) and sick sinus syndrome (*N* = 1, 10%). PPM implantation after coronary artery bypass graft (CABG) surgery was the lowest (0.63%), while combined CABG and valve operations had the highest incidence (5.97%). Independent risk predictors for PPM implantation included female gender (*p* = 0.03), rheumatic heart disease (*p* = 0.008), pulmonary hypertension (*p* = 0.01), redo operations (*p* = 0.002), mitral valve procedures (*p* = 0.001), tricuspid valve procedures (*p* = 0.0003) and combined mitral and tricuspid valve procedures (*p* = 0.0001). Average length of intensive care unit (ICU)/high-dependency unit (HDU) stay was significantly prolonged for patients who required a PPM post-cardiac surgery.

**Conclusion:**

As clinicians, it can be challenging to provide our patients with accurate information on the risk of PPM implantation relative to their operation. A unit-specific data may be a more accurate method of informing our patients on this risk.

## Introduction

The prevalence of permanent pacemaker (PPM) implantation following cardiac surgery had been reported to be between 0.4 and 6%, with the lowest incidence following coronary artery bypass grafting (CABG) and the highest following valve surgery [1, 2]. Common indications for PPM post-cardiac surgery include bradyarrhythmias, such as atrioventricular (AV) block, sinus node dysfunction and atrial fibrillation (AF) with a slow ventricular response [3]. These arrhythmias can occur secondary to myocardial injury that is sustained during the operation, resulting in damage to the conduction system of the heart. The pathophysiological mechanisms causing perioperative myocardial injury include mechanical trauma, which can arise during valve operations where there is direct contact with the conducting system, and ischaemic injury, which can arise following any cardiac procedures due to inadequate intra-operative blood supply to the myocardium [4].

Furthermore, arrhythmias that require pacing can occur as a result of pre-existing, undiagnosed structural or electrophysiological abnormalities. Hence, previous studies have identified various preoperative risk factors associated with PPM dependency, such as advanced age, female gender, redo operations, prior valve surgery and pre-existing conduction abnormalities, PR interval > 200 ms or left bundle branch blocks (LBBB) [2, 5]. Identification of those at high risk of conduction abnormalities would allow clinicians to accurately consent patients for potential PPM insertion and may help establish novel surgical techniques that protect the conduction system in such patients.

With the advancement of technology, minimally invasive and transcatheter modalities have provided an additional option for patients undergoing valve interventions. Transcatheter aortic valve implantation (TAVI) has, in recent years, gained popularity in patients at intermediate-to-high or prohibitive surgical risk [6, 7]. While studies have shown TAVI to be a noninferior alternative to surgical aortic valve replacements (AVR), the PPM rates post-TAVI have been reported to be significantly higher, reaching percentages as high as 34% [7, 8] versus 2–8% in surgical AVR [9, 10]. TAVI is slowly being extended to low-risk patient cohorts. PPM implantation is therefore a complication to consider if surgery is an option and accurate figures regarding the risk of PPM dependency is important.

In our study, we aim to evaluate the incidence of PPM insertion in a single tertiary cardiothoracic centre, examining our contemporary PPM implantation rates relative to those reported in the literature. We also aim to identify the risk factors for PPM insertion in our cohort of patients and its impact on our patients’ length of intensive care unit (ICU)/high-dependency unit (HDU) stay.

## Methods

### Patient characteristics

A retrospective analysis of 403 patients was carried out from October 2018 to April 2019 in the cardiothoracic unit of the Royal Victoria Hospital, Belfast. Patients’ data were obtained from the Northern Ireland Electronic Care Record (NIECR), and all operative data was obtained from a hospital database (Dendrite Clinical System). Exclusion criteria included previous PPM or implantable cardioverter defibrillator (ICD), patients with congenital heart disease, patients who had an indication for PPM implantation prior to cardiac surgery and intraoperative or postoperative death during hospital stay, with a cutoff of 15 days post-cardiac surgery.

### Operative data

In our study we included all CABG operations, CABG and valve operations, valve only operations and other operations including modified Bentall’s procedure, excision of atrial myxomas, personalized external aortic root support (PEARS) procedure, repair of rupture of left atrial appendage, pericardiectomy and the repair of aortic dissection and aneurysm. All redo and emergency operations were included in this study. We also compared the average length of ICU and HDU stay and the average length of postoperative stay. All operations were carried out through a median sternotomy with the exception of 11 mini-sternotomy AVR and four transapical aortic valve implantations.

### Statistical analysis

Prism 7 software was used to analyse the data. Continuous variables were expressed as mean ± standard deviation, and categorical variables were summarized as frequencies and percentages. To compare continuous variables, the Student’s independent *t* test was used, while Fisher’s exact test was used to compare categorical variables. Statistical significance was defined as *p* value < 0.05. A stepwise logistic regression analysis was performed to identify predictors of PPM.

## Results

Four hundred and three patients were included in this study. Mean age of the study population was 66 ± 10 years. Ten patients required a PPM post-surgery (2.48%), four of whom were male (40%) and six were female (60%). Table 1 demonstrates the comparison between the demographic variables and preoperative comorbidities of the PPM group and non-PPM group. Significant risk factors for PPM implantation post-cardiac surgery included rheumatic heart disease (RHD) (*p* = 0.008) and pulmonary hypertension (*p* = 0.01). Preoperative electrocardiographic (ECG) characteristics, left ventricular ejection fraction (LVEF) and New York Heart Association (NYHA) classes were also compared between the two groups. None of these variables demonstrated statistical significance.

**Table 1:** Comparison of demographic variables and preoperative comorbidities between non-PPM group and PPM group

	Non-PPM	PPM	*p* value
*N*	393	10	
Demographic data			
Age	66 ± 10 years	66 ± 16 years	> 0.9
Female	110 (28%)	6 (60%)	0.04*
Rheumatic heart disease	4 (1%)	2 (20%)	0.008*
Coronary artery disease	211 (54%)	5 (50%)	> 0.9
Hypertension	223 (57%)	5 (50%)	0.75
Diabetes	116 (30%)	2 (20%)	0.7
Hypercholesterolaemia	112 (28%)	0 (0%)	0.07
PVD	20 (5%)	1 (10%)	0.4
CKD	36 (9%)	3 (30%)	0.06
COPD	27 (7%)	1 (10%)	0.5
Pulmonary hypertension	6 (2%)	2 (20%)	0.01*
Preoperative heart rhythm			
Sinus rhythm	334 (85%)	8 (80%)	0.7
AF	56 (14%)	2 (20%)	0.6
Bundle branch blocks	1 (0.3%)	0	> 0.9
1st degree AV block	1 (0.3%)	0	> 0.9
VF/VT	1 (0.3%)	0	> 0.9
Left ventricular ejection fraction (LVEF)			
Good > 50%	319 (81%)	8 (80%)	> 0.9
Moderate 31–50%	61 (16%)	2 (20%)	0.65
Poor 21–30%	10 (3%)	0 (0%)	> 0.9
Very poor < 21%	3 (1%)	0 (0%)	> 0.9
NYHA class			
I	62 (16%)	0 (0%)	
II	226 (58%)	5 (50%)	0.2
III	93 (24%)	5 (50%)	
IV	12 (3%)	0 (0%)	

*PVD* peripheral vascular disease, *CKD* chronic kidney disease, *COPD* chronic obstructive pulmonary disease, *AF* atrial fibrillation, *VF/VT* ventricular fibrillation/ventricular tachycardia, *NYHA* New York Heart Association

The comparison of operative data between our non-PPM group and PPM group is demonstrated in Table 2. A total of 159 CABG operations were performed with one patient requiring a pacemaker (0.6%), making it the lowest percentage of PPM implantation post-procedure, while combined CABG and valve operations had the highest pacemaker implantation rates (5.9%). A total of 161 valve-only operations were performed with five requiring a pacemaker post-surgery (3.1%). Of all the valvular procedures, mitral valve (MV) (*p* = 0.001) and tricuspid valve (TV) procedures (*p* = 0.0003) were significant risk factors for PPM implantation post-procedure. The risk was significantly raised when both these valves were operated on simultaneously (*p* = 0.0001).

**Table 2:** Comparison of operative data between non-PPM group and PPM group

Variables	Non-PPM	PPM	Percentage (%)	*p* value
CABG	158 (40%)	1 (10%)	0.63	0.1
CABG + valve operations	63 (16%)	4 (40%)	5.97	0.07
Valvular procedures (without CABG)	156 (40%)	5 (50%)	3.11	0.5
AVR-related procedures	172 (44%)	5 (50%)	2.82	0.8
Isolated AVR	96 (24%)	1 (10%)	1.03	0.5
MV-related procedures	47 (12%)	6 (60%)	11	0.001*
TV-related procedures	12 (3%)	4 (40%)	25	0.0003*
MV + TV related procedures	8 (2%)	4 (40%)	33	0.0001*
Mini-sternotomy	11 (11%)	0 (0%)	0	> 0.9
Redo operations	8 (2%)	3 (30%)	27	0.0016*
Emergency operations	2 (1%)	1 (10%)	33	0.1
Average length of ICU/HDU stay (days)	4 ± 7 days	15 ± 14 days		0.03*
Average length of postoperative stay (days)	13 ± 13 days	23 ± 16 days		0.08

*CABG* coronary artery bypass graft, *AVR* aortic valve replacement, *MV* mitral valve, *TV* tricuspid valve, *ICU* intensive care unit, *HDU* high-dependency unit

We note a low post-AVR PPM implantation rate of 1.03% in our study. There were eleven patients who underwent AVR through a mini-sternotomy incision, none of whom required a PPM. This was not statistically significant when compared with the patients who underwent AVR through a median sternotomy incision.

All operations were carried out on an elective basis with the exception of three which were carried out as emergency procedures. Redo operations were performed in eleven patients, all of which were redo valvular procedures. Three patients required a PPM post-surgery (*p* = 0.002), two of which had combined MV and TV procedures performed and one patient underwent AVR. Average length of ICU/HDU stay was significantly longer for patients who required a PPM, with a mean stay of 15 ± 14 days, compared with 4 ± 7 days for patients who did not require a PPM (*p* = 0.03). The average length of postoperative stay was 13 ± 13 days in patients who did not require a PPM compared with 23 ± 16 days in patients who required a PPM (*p* = 0.08). The most common indication for PPM implantation in our patient cohort was complete heart block (*N* = 7, 70%). This was followed by bradycardia/pauses during sustained atrial tachycardia (AT)/atrial flutter (AFL)/atrial fibrillation (AF) (*N* = 2, 20%) and sick sinus syndrome (*N* = 1, 10%). Although complete heart block was observed more frequently after AVR-related procedures, there was insufficient statistical significance to prove a correlation.

## Discussion

Our single-centre PPM implantation rate after cardiac surgery of 2.48% was found to be consistent with the rates quoted by literature as far back as 1984 by Gordon et al. [1]. In their retrospective review of 10,421 patients, 2.4% required PPM postoperatively. We also found our data to be comparable with that of other studies that categorized surgical procedures to CABG, CABG and valve and valve-only operations. A literature review of 24,729 patients found overall post-operative PPM requirement to be 2.2% versus 2.48% in our study, 0.8% for CABG operations versus 0.63% in our study and 3.5% for valve-only operations versus 3.11% in our study [11]. These statistics demonstrate that PPM implantation rates after cardiac surgery, irrespective of the type of surgery, have remained largely unchanged over the past three decades despite improvement in surgical technique and technological advancements. This suggests that individual patient risk factors may outweigh surgical factors when predicting the need for a PPM.

Univariate analysis of demographic variables and preoperative comorbidities of our patients found female gender, RHD and pulmonary hypertension to be significant risk factors for postoperative PPM dependency. While RHD is a well-recognized cause for arrhythmias, to our knowledge, this is the first study to find an association between RHD and an increased need for PPM post-cardiac surgery. Although many studies have recognized pulmonary hypertension and tricuspid valve operations as risk predictors for PPM insertion post-cardiac surgery, it is possible that RHD is an independent risk factor. RHD typically causes mitral incompetence in the early stages. Persistent valvulitis of the mitral valve leads to bicommissural fusion, resulting in mitral stenosis [12]. Over time, pulmonary hypertension ensues causing right ventricular dilatation and tricuspid regurgitation. Hence, a patient with RHD often presents with multiple valvular pathologies and myocarditis, placing them at significant risk of cardiac conduction abnormalities before and after surgery. While RHD has previously been reported to be more frequently found in women [13, 14], other studies have found female gender to be an independent risk factor for PPM implantation post-cardiac surgery [2, 15].

The requirement for PPM insertion is more frequent after valve surgery than most other cardiac procedures. This may be due to the close proximity of the valves to the conduction system of the heart. Our study found the incidence of PPM implantation after any MV- and TV-related procedures to be higher than other valvular interventions. When the mitral and tricuspid valves are operated on simultaneously, the risk of PPM implantation significantly increases. The septal cusp of the tricuspid valve forms one of the borders of the triangle of Koch, which contains the atrioventricular node and its penetrating bundle. As the bundle passes through the apex of the triangle directly into the left ventricular outflow tract, it has close anatomical relations to the tricuspid and mitral valves, placing it at risk during valve surgery. Jouan et al. found that 14.5% of patients who underwent isolated mitral valve surgery developed high-grade cardiac conduction disorders lasting longer than 3 days postoperatively. When concomitant tricuspid valve ring annuloplasty was carried out, the occurrence increased to 41.2% [16]. While their study did not evaluate the PPM implantation rates post-surgery, another study quoted a PPM implantation rate post-tricuspid valve procedure of 21% [15].

The tricuspid valve is rarely operated upon in isolation. Secondary tricuspid regurgitation as a result of mitral valve disease or ischaemic left ventricular failure is often the pathology requiring surgical intervention. Hence, when a tricuspid valve operation is justified, the cohort of patients often have biventricular cardiac failure and pulmonary hypertension. Pulmonary hypertension has long been established as an independent cause for arrhythmias, particularly supraventricular arrhythmias [17]. Expectedly, the risk of PPM implantation becomes greatly heightened due to a defective conduction system prior to surgery. The anatomical factors of valvular surgery and patient comorbidities may also explain our observation of redo valve operations as a significant risk factor for PPM implantation, a finding consistent with other studies that have demonstrated both immediate and longer-term PPM dependency [1, 11].

Our PPM rate post-isolated AVR of 1.03% was found to be relatively lower than that reported in other studies [9, 10]. The aortic valve is the most common valve to be operated on due to its predisposition to developing calcification with ageing. The calcification of the valve leaflets and its surrounding annulus affects the nearby atrioventricular node and bundle of His. Surgical manipulation during AVR operations can lead to further mechanical trauma to the conduction system, generating new conduction defects or exacerbating pre-existing ones. The low PPM post-AVR rates may be attributable to improved surgical technique or perhaps the use of TAVI in patients with intermediate risk. This may have resulted in a patient cohort that is relatively low risk with fewer pre-existing conduction disturbances.

In the current era, minimally invasive approaches are becoming more favourable, including valve replacements. TAVI is increasingly used and is slowly being extended to low-risk patients. Similarly, the use of sutureless valves has been gaining popularity since it reduces overall duration of procedure and cardiopulmonary bypass and aortic cross-clamp times. However, these procedures are associated with significantly elevated rates of PPM insertion, 25.9% for TAVI versus 6.6% for surgical AVR [7] and 8.8% for rapid deployment valves versus 3.7% for surgical AVR [18]. These rates have remained high despite the introduction of the latest generation transcatheter valve systems and evolution of sutureless valve technology, but we recognize that the high observed rates might be confounded by the use of these modalities in high-risk cohorts with multiple cardiac comorbidities and conduction disturbances.

PPM insertion is not without its risks and complications. In the acute phase, patients can develop wound infections and pocket infections, pocket haematoma and issues with the pacemaker device such as lead dislodgement or dysfunction, pocket fibrosis, pneumothorax and myocardial rupture. After cardiac surgery, PPM insertion has been associated with prolonged HDU and ICU admissions, which by themselves carry high risks of morbidity and mortality [1]. Progression of heart failure and AF in PPM-dependent patients have also been reported, increasing long-term mortality risks [19].

A risk predictor model was designed by Koplan and colleagues in 2003 to risk stratify patients who may require a PPM after cardiac valve surgery [5]. In their model, right bundle branch blocks (RBBB) and tricuspid valve operations were strong independent risk factors. Additional preoperative characteristics such as left bundle branch blocks (LBBB), PR interval > 200 ms, prior valve surgery, age > 70 years and other multivalve surgeries also posed as risk factors in their model. While similarities in risk factors for PPM implantation post-cardiac surgery have been drawn across countless studies, the reliability of a risk predictor model is questionable. For several reasons: firstly there is little evidence of the use of this model in clinical practice; secondly PPM rates differ across hospitals and patient cohorts and are largely dependent on various factors including surgical technique, type of operation and patients’ preoperative characteristics; and thirdly inconsistencies in risk factors have been reported across studies such as the relevance of age and gender. The use of unit specific data may be a more accurate method of informing our patients of the risk. Furthermore, having this knowledge can assist surgeons in decision-making and potentially modify their surgical approach when operating on patients with multiple risk factors.

Major limitations of our study were its retrospective nature and the small cohort of patients included in this study. As a single-centre study, we cannot generalize our results to other hospital settings. The demographics and socioeconomic status of our population may differ from patients elsewhere. Therefore, it would be interesting to confirm our findings in a larger multicentre trial. Subtle conduction abnormalities may have been recorded as an ECG in sinus rhythm, due to the limitations of our database. Finally, peri- and postoperative use of atrioventricular (AV) blocking drugs was not recorded in our study. Their use had been reported to be potential risk factors for PPM implantation and may be a confounding factor that was not considered in our analysis [20].

## Conclusion

While the rates of PPM implantation post-cardiac surgery have remained stable over the past decades, our study has shown a slight improvement in PPM implantation rates post-AVR. This should be taken into consideration in an era where TAVI is a treatment option. PPM implantation is not without its risks and complications. Our study has demonstrated a prolonged ICU/HDU admission associated with PPM implantation after surgery, which itself has been linked to an increase in morbidity and mortality. As clinicians, it can be challenging to provide our patients with accurate information on the risk of PPM dependency after cardiac surgery. Hence, a unit-specific data may be a more accurate method of informing our patients on this risk. The independent risk factors for our unit include female gender, rheumatic heart disease, pulmonary hypertension, redo operations and mitral and tricuspid valve procedures.

## Data Availability

The authors affirm that this manuscript is an honest, accurate and transparent account of the study being reported. No important aspects of the study have been omitted, and any discrepancies from the study as planned have been explained.
